# A usability study on the inGAIT-VSO: effects of a variable-stiffness ankle-foot orthosis on the walking performance of children with cerebral palsy

**DOI:** 10.1186/s12984-024-01433-7

**Published:** 2024-08-01

**Authors:** Luc van Noort, Nikko Van Crey, Elliott J. Rouse, Ignacio Martínez-Caballero, Edwin H. F. van Asseldonk, Cristina Bayón

**Affiliations:** 1 Centro de Automática y Robótica, Consejo Superior de Investigaciones Científicas, CAR-CSIC-UPM, Madrid, Spain; 2 Department of Biomechanical Engineering, University of Twente, Enschede, The Netherlands; 3 Department of Robotics, University of Michigan, Michigan, United States; 4 Department of Mechanical Engineering, University of Michigan, Michigan, United States; 5 Hospital Infantil Universitario Niño Jesús, Madrid, Spain

**Keywords:** Ankle-foot orthosis, Ankle stiffness, Cerebral palsy, Gait, Assistive technology

## Abstract

**Background:**

Ankle-foot orthoses (AFOs) are commonly used by children with cerebral palsy (CP), but traditional solutions are unable to address the heterogeneity and evolving needs amongst children with CP. One key limitation lies in the inability of current passive devices to customize the torque–angle relationship, which is essential to adapt the support to the specific individual needs. Powered alternatives can provide customized behavior, but often face challenges with reliability, weight, and cost. Overall, clinicians find certain barriers that hinder their prescription. In recent work, the Variable Stiffness Orthosis (VSO) was developed, enabling stiffness customization without the need for motors or sophisticated control.

**Methods:**

This work evaluates a pediatric version of the VSO (inGAIT-VSO) by investigating its impact on the walking performance of children with CP and its potential to be used as a tool for assessing the effect of variable stiffness on pathological gait. Data was collected for three typical developing (TD) children and six pediatric participants with CP over two sessions involving walking/balance tasks and questionnaires.

**Results:**

The sensors of the inGAIT-VSO provided useful information to assess the impact of the device. Increasing the stiffness of the inGAIT-VSO significantly reduced participants’ dorsiflexion and plantarflexion. Despite reduced range of motion, the peak restoring torque increased with stiffness. Overall the participants’ gait pattern was altered by reducing crouch gait, preventing drop-foot and supporting body weight. Participants with CP exhibited significantly lower (p < 0.05) physiological cost when walking with the inGAIT-VSO compared to normal condition (own AFO or shoes only). Generally, the device did not impair walking and balance of the participants compared to normal conditions. According to the questionnaire results, the inGAIT-VSO was easy to use and participants reported positive experiences.

**Conclusion:**

The inGAIT-VSO stiffnesses significantly affected participants’ plantarflexion and dorsiflexion and yielded objective data regarding walking performance in pathological gait (e.g. ankle angle, exerted torque and restored assistive energy). These effects were captured by the sensors integrated in the device without using external equipment. The inGAIT-VSO shows promise for customizing AFO stiffness and aiding clinicians in selecting a personalized stiffness based on objective metrics.

## Background

Cerebral palsy (CP) encompasses a range of neurological disorders arising from damage during birth or early development, resulting in lasting impairments in strength, muscle function, balance, and posture control [1]. Globally, there are approximately 18 million individuals living with CP [2].

Mobility is an integral part of development in children [3, 4]. However, children with CP often experience higher energy expenditure during walking compared to those with a typical development (TD). The ankle joint plays a crucial role in the efficient and proper progression of the body in gait [4]. Notably, children with CP exhibit a major muscle dysfunction in the distal joints of the lower extremities, which puts ankle-foot orthoses (AFOs) among the primary assistive devices prescribed for these individuals [4–6].

While traditional AFOs have provided many benefits, they typically lack in mimicking the dynamics, energy cycling, or impedance of the human ankle joint [6–11]. Instead, these traditional solutions normally lock the ankle in place to maintain ground clearance during leg swing, and ensure that each stride begins with a heel strike instead of flat-footed. Thus, their adaptability to individual needs is compromised due to the maintenance of a static stiffness throughout the entire gait cycle. In addition to these limitations, clinicians face further challenges in prescribing the ideal orthotic management tailored to specific individual needs [6, 10, 11]. First, current clinical standards for selecting the AFO stiffness and its impact on patient performance remain unclear [10–13]. Second, evaluating a patient’s walking performance is time-consuming and requires sophisticated equipment limited to a controlled environment [14].

New passive and powered assistive AFOs have been developed to address some of the shortcomings of traditional AFOs, but they are still limited in function and practicality to be applied in a heterogeneous population as pediatric patients with CP [8, 10]. Passive assistive solutions hold a distinct advantage over powered counterparts by eliminating the need for motors and batteries, resulting in reduced weight, complexity, and cost. Consequently, a majority of the commercially available assistive AFOs are passive, such as the Neuro Swing (Fior &Gentz, Holly Springs, NC, USA), Nexgear Tango (Ottobock, Duderstadt, Germany), and Ultraflex AFO Joint (Ultraflex Systems, Pottstown, PA, USA). There are also several research prototypes, normally intended for adults, that strive to assist the user through passive mechanisms [8, 15–18]. Unfortunately, the majority of these research/commercial systems are too heavy for pediatric patients (>1 kg) or cannot customize the shape of the torque–angle relationship to specific user’s needs and capabilities, which in CP change with age and stage of rehabilitation [7, 8, 15–17].

In recent work, Van Crey et al. [19] introduced the design of the Variable Stiffness Orthosis (VSO), a quasi-passive AFO that allows for customizable torque–angle relationships and step-to-step motorized adjustment of its average stiffness. The features of the VSO are promising for the heterogeneous pediatric population with CP. The AFO stiffness significantly affects pathological gait [7], and we envision that, when selected properly, the customization of this stiffness can help patients with CP to walk more naturally [7, 10, 17]. Additionally, with these features, clinicians could also quickly assess the immediate effects of different stiffness conditions on the specific patient’s performance and adapt accordingly for the unique needs of each patient, which would aid in AFO prescription [6, 10, 11].

In this work, we evaluate the effects of a pediatric version of the VSO [19] (inGAIT-VSO) on the gait of children with CP without prior training. This evaluation aims to assess the impact of the inGAIT-VSO on CP gait kinematics and its potential effectiveness as as tool for collecting objective data on pathological gait, which could aid in AFO prescription. We also examine participants’ acceptance and usability of the inGAIT-VSO with questionnaires. In the following sections we describe the inGAIT-VSO and present the results of testing with both pediatric participants with CP and TD.

## Methods

### Prototype overview

#### Variable stiffness orthosis

**Fig. 1:**
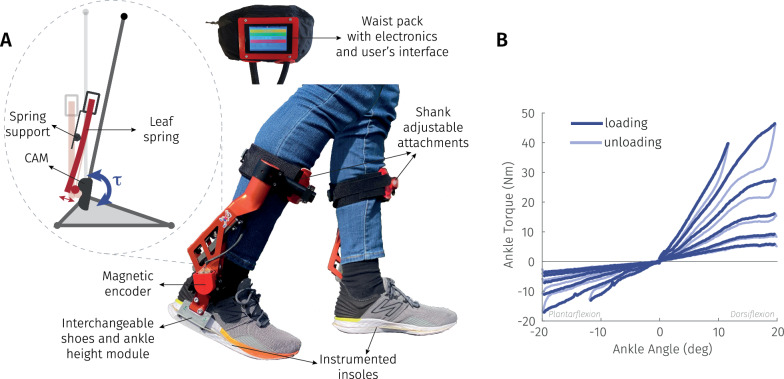
**A** Illustration of the inGAIT-VSO orthosis indicating the main components and a scheme of the passive element working principle. **B** Example of torque–angle curves extracted from the characterization of the device. Six curves are shown out of eleven tested. The difference between loading and unloading represents the energy loss

The inGAIT-VSO (Fig. 1A) is a pediatric version of the Variable Stiffness Orthosis (VSO) developed by Van Crey et al. [19]. Unlike Van Crey et al., the inGAIT-VSO is targeted towards children with CP and features a decreased height to accommodate smaller shank lengths, decreased torque output, and manual (rather than motorized) adjustment of ankle stiffness (Table 1). These design trade-offs allowed for a decreased mass suitable for children, but the inGAIT-VSO is otherwise identical to the VSO and a more detailed description of its operating principles can be found in [19].

During gait, the ankle joint rotates and a cam deflects a leaf spring, producing a restorative torque about the ankle joint. The cam profile is an interchangeable component that governs the shape of the torque–angle relationship (Fig. 1B). By specifying the shape of the cam profile, the torque–angle relationship can be customized for different individuals and pathologies. The stiffness magnitude can also be adjusted according to user/clinician preferences by manually repositioning a spring support (via lead screw) (Fig. 1A), effectively rotating the torque–angle relationship about the origin. These adjustments are made when the ankle-joint is offloaded and can be made quickly without replacing components. The stiffness impacts both plantarflexion and dorsiflexion movements from the neutral position, preventing drop-foot during swing and supporting body weight during stance, with a restoring torque that contributes to ankle push-off.

#### Characterization with custom dynamometer

Mathematical models were used to generate a cam profile that renders a customized torque–angle relationship [20] and to simulate the torque–angle relationships available at different spring support locations [19].

The model predictions were empirically verified with the Neurobionics Lab Rotary Dynamometer [19]. The inGAIT-VSO was mounted to the dynamometer and actuated in both dorsiflexion (DF) and plantarflexion (PF) at a mean angular velocity of 5 deg/s. A rotary encoder and load cell (45E15A M63J, JR3, Inc., Woodland, CA, USA) on the dynamometer collected torque and device angle at 1 kHz. The torque data were low-pass filtered with a 2 Hz passband frequency. The torque–angle relationships were measured at eleven stiffness conditions equally spaced between the softest and the stiffest settings (Fig. 1B).

The torque–angle relationship in PF and DF can be designed independently. In this study, we designed the cam profile to be linear in early stance and three times stiffer in DF than PF. In DF the torque–angle relationship was also designed to soften past $\sim$ 12 deg to allow forward progression during controlled DF. In dynamometer testing the average stiffness ranged from 0.39–3.43 Nm/deg towards DF, and 0.28–1.16 Nm/deg towards PF. The average stiffness in controlled DF can be modulated by a factor of 8.8 (maximum stiffness divided by the minimum stiffness), with an average energy efficiency of 86% (Fig. 1B).

#### Electronics and data capturing

The inGAIT-VSO was sensorized to directly capture the effects of the stiffness without needing sophisticated lab equipment. Concretely, ankle angle is recorded with a magnetic encoder (AS5048b, AMS-OSRAM AG, Premstaetten, Austria) and ground contact with two force sensitive resistors (FSR) embedded within the insole (FlexiForce A502, Tekscan Inc, MA, USA). The FSRs were used to detect heel strike and toe off gait events. The data were logged at 100 Hz using a microcontroller (Orange Pi Zero3, Shenzhen Xunlong Software Co., Ltd, Shenzhen, China). A graphical user interface was developed in Python which facilitated sensor data logging and user operation [21]. All electronics were carried out by the user in a waist pack and operated with a maximum voltage of 5 V.

**Table 1:** Main technical features of the inGAIT-VSO

Type of device	Passive
Weight (kg)	0.5–1 depending on the size
Stiffness range DF (Nm/deg)	0.39–3.43
Stiffness range PF (Nm/deg)	0.28–1.16
Range of motion (deg)	Adaptable, with a maximum of 35 DF, 30 PF, 15 AI and 75 AE
Max voltage (V) used by electronics	5
Data logging frequency (Hz)	100
Battery life with touchscreen on (h)	3.5
Recommended user’s height (m)	1.1–1.75
Maximum user’s weight (kg)	60

*DF* dorsiflexion, *PF* plantarflexion, *AI* ankle inversion, *AE* ankle eversion

### Study design

#### Experimental protocol

A descriptive experimental study was performed to evaluate participants’ acceptance and immediate effects on walking performance when using the inGAIT-VSO without prior training. The Local Ethical Committee of Hospital Infantil Universitario Niño Jesús (HNJ) gave approval to the study (R-0064/23) and warranted its accordance with the Declaration of Helsinki. All participants and families were informed, and parental consents were obtained prior to participation.

**Fig. 2:**
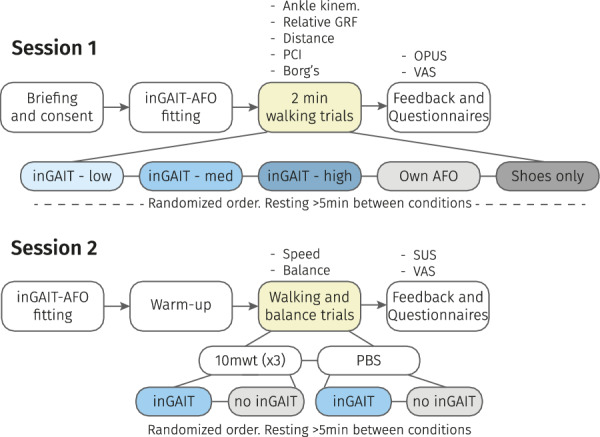
Schematic depiction of the experimental protocol

The study involved two sessions which took place on two different days at the HNJ (Fig. 2). The first session of the study consisted of four phases. The participants were assisted in putting on the inGAIT-VSO and were allowed some time to walk around with it to experience the device and report any discomfort. After this, the two-minutes walking test (2mwt) [22] was performed in five different conditions: wearing the inGAIT-VSO with (1) low stiffness (0.2 Nm/kg, as peak restoring torque at 12 deg of DF), (2) medium stiffness (0.4 Nm/kg at 12 deg of DF), and (3) high stiffness (0.6 Nm/kg at 12 deg of DF), (4) wearing their own AFO (if they used one), and (5) with shoes only. The values for the peak restoring ankle torque were selected to be consistent with and in the range of the literature [7, 15, 16], and the 12 deg of DF approximately corresponded to the maximum DF during stance of a healthy gait [23]. The human attachments were adjusted such that zero ankle torque (neutral position) occurs when the shank is perpendicular to the foot. The same shoes were used in all conditions. The order of the testing conditions was randomized between participants, and after each 2mwt, participants could rest as long as needed before passing to the next walking condition.

For each condition, the distance walked and the heart rate were measured, as well as the sensor data retrieved from the inGAIT-VSO (if worn). Part of this information was later used to calculate the physiological cost index (PCI) [24, 25], a metric that combines speed and heart rate in a single index:

1 $$\begin{aligned} PCI = \frac{HR_{w}-HR_{r}}{s} \end{aligned}$$

where $HR_{r}$ and $HR_{w}$ are the heart rate measurements (beats/min) at resting and immediately after finishing the 2mwt, respectively, and *s* is the average walking speed during the 2mwt (m/min).

Participants also reported the effort perceived during the 2mwt using the Borg’s scale [26] and any pain or discomfort was noted down using the VAS scale [27]. Finally, participants completed a modified version of the OPUS questionnaire in its module of “Satisfaction with Device” [28] to report (using a 5–point Likert scale, ranging from 1—strongly disagree to 5—strongly agree) their experience, acceptance, and other feelings related to the use of the inGAIT-VSO (Fig. 2).

At the end of the first session, the best performing stiffness with the inGAIT-VSO was selected to be used in the second session. For this selection we considered three variables: distance walked, PCI, and Borg’s scale reported by participants. Considering the three levels of stiffness tested, each variable was assigned a numeric score (1, 2, or 3) based on performance, with higher scores indicating larger outcomes for distance walked, and lower outcomes for PCI and Borg’s scale. The final selection of the best performing stiffness condition was determined by applying the formula $0.1 \cdot D + 0.6 \cdot PCI + 0.3 \cdot B$, where *D*, *PCI*, and *B* represent the scores for distance, PCI, and Borg’s scale, respectively. The weights chosen were based on our logical reasoning, and additional argumentation can be found in the Discussion section.

The second session of the study also consisted of four phases (Fig. 2). During the preparation and warm-up, participants were given the time to walk around a bit with the inGAIT-VSO configured with the best performing stiffness level selected after the first session. Then, participants were asked to perform the ten-meters walking test (10mwt) [29] both with the inGAIT-VSO in the selected best performing configuration and with their own non-assistive AFO if they used one, or with shoes only otherwise. The order of these conditions was randomized between participants. Subsequently, the pediatric balance scale (PBS) [30, 31] was followed to evaluate the balance capabilities with and without the inGAIT-VSO. It was assessed by two researchers involved in the study (LvN, CB), taking as final score of each PBS task the average of the results reported by both researchers individually. Finally, the System Usability Scale (SUS) [32] was completed by the participants to provide a measure of their perception of the usability (Fig. 2).

#### Participants

Nine participants (3 TD, 6 CP) took part in the study (age 9.67 ± 1.63 years-old, weight 29.44 ± 4.60 kg, height 1.37 ± 0.06 m) (Table 2). TD participants were included to pilot the inGAIT-VSO and the protocol prior to trying with CP, and to assess the effects of the device on able-bodied children. TD performed the full protocol except the PBS tests on the second session, as we assumed those tasks were fairly simple for this group. The inclusion criteria for participants’ recruitment followed:

- Participants with CP: children aged 5-17 diagnosed with (predominantly) spastic uni/bilateral CP, gross motor function levels I-III, sufficient cognitive skills, and capable to follow the protocol walking time. Additionally, flexible equinus deformities or drop-foot were desired, with Ashworth scale scores between 1 and 3.
- Participants with TD: children aged 5-17 showing a TD who could follow the protocol walking time.

The exclusion criteria were: recent leg surgery or Botulin toxin A injections, musculoskeletal deformities or unhealed skin lesions hindering device use, gastrocnemius shortening >10 deg, visual deficits affecting protocol performance, behavioral issues hindering cooperation, comorbidity affecting walking, visual, or cognitive abilities, and dyskinetic predominance.

**Table 2:** Participants characteristics

Participant ID	Age (years-old)	Weight (kg)	Height (m)	Shoe size (EU)	Pathology	Pattern	GMFCS	Sex	Own AFO	External aid	inGAIT-VSO weight (kg)
TD01	12	39	1.51	38	Healthy	–	–	Male	–	–	0.85
TD02	7	24	1.30	32	Healthy	–	–	Female	–	–	0.7
TD03	10	31	1.37	35	Healthy	–	–	Female	–	–	0.7
CP01	8	30	1.32	33	Hemiparesia (left)	Equinus	I	Male	Yes	–	0.7
CP02	11	25	1.34	36	Spastic diplegia	Crouch	II	Female	Yes	–	0.8
CP03	9	25	1.35	32	Spastic diplegia	Crouch	III	Female	No	–	0.7
CP04	11	27	1.37	35	Spastic diplegia	Crouch	II	Male	Yes	Walker	0.7
CP05	11	34	1.41	37	Hemiparesia (right)	Jump gait	I	Male	Yes	–	0.85
CP06	9	30	1.34	35	Hemiparesia (left)	Equinus	I	Male	No	–	0.7

The weight of the inGAIT-VSO includes the shoe, so it varied depending on the shoe size utilized

### Data analysis

Data analysis included sensor data from the AFO, participants’ performance in the different tests and responses to the OPUS and SUS surveys. Additionally we noted down participants’ capacity to finish the two sessions of the protocol without the occurrence of adverse events and completing the different tasks (2mwt, 10mwt, PBS) while utilizing the inGAIT-VSO.

#### Data processing

Ankle joint angles and pressure forces derived from the sensors of the inGAIT-VSO were processed using Matlab 2023b (Mathworks, Natick, MA, USA). Left heel strike events that occurred during the 2mwt were used to identify gait cycles. Each gait cycle was linearly spline interpolated to the same number of data points. Mean profiles and standard deviation (SD) as a function of the gait cycle were computed for each 2mwt condition. Ankle kinematics were used to assess the effects of the different stiffness levels with the inGAIT-VSO. Maximum ankle DF and PF were obtained for each gait cycle.

Exerted ankle torques for each gait cycle performed in the 2mwt were computed based on the model of the inGAIT-VSO in the specific tested configuration and the joint angles measured with the encoders. Mean torque profiles were determined for each stiffness level with the inGAIT-VSO.

Stored energy towards DF (loading) was calculated as the integral of the torque–angle relationship for each stance phase performed during the 2mwt. For each stored energy during stance we also computed the energy loss as the difference between loading (stored) and unloading (release) paths. Finally, mean stored and loss energies during stance phase were computed for each stiffness level, deriving the mean energy efficiencies [(stored-loss)/stored] per stiffness level.

Other outcomes like distances walked, time expended, effort perceived, PBS, OPUS and SUS were noted down by two experimenters (LvN, CB) and postprocessed using Matlab 2023b. For the SUS scale, we considered a SUS score between 0–21 as “worst imaginable”, between 22–39 as “poor”, 40–52 as “ok”, 53–73 as “good”, 74–85 as “excellent” and 86–100 as “best imaginable”, in terms of usability [33].

#### Statistics

Mean and SD were the main descriptive statistics used to summarize the characteristics of data samples. The normality of distribution was checked using the Kolmogorov-Smirnov test. The small sample size and the large heterogeneity made that the criteria for normality were not met for any of the variables tested.

To identify the effects of the three stiffness levels of the inGAIT-VSO on ankle kinematics, we executed the Friedman test using as variables of interest the mean maximum DF and PF achieved during the 2mwt for the six participants with CP. Our hypothesis was that a higher stiffness would lead to lower degrees of maximum ankle DF and maximum PF. If the Friedman test returned a significant effect, post-hoc analysis was performed in the form of Wilcoxon signed-rank tests.

Subsequently, we assessed the effect of the inGAIT-VSO on the outcomes of the 2mwt (distance and PCI), the 10mwt (speed) and the PBS for participants with CP. To do that, we used the Wilcoxon signed-rank test to compare the best performing stiffness of the inGAIT-VSO to the participant’s normal walking condition (shoes only or their own AFO if applicable).

An alpha level of 0.05 was used in all statistical tests.

## Results

All participants completed the study without problems and no adverse events or skin integrity issues were detected. Participants TD01 and TD02 reported device-related little to moderate pain (levels 2 and 4 of the VAS scale) after session 1 of the study. All other participants experienced no pain while using the inGAIT-VSO. No participant experienced excessive fatigue after the training.

Between the first and second session, the selection of the best performing condition with inGAIT-VSO resulted in Low stiffness for two participants (CP04 and CP05), and Medium stiffness for four participants (CP01, CP02, CP03 and CP06).

In the following sections, we focus on analysis for participants with CP, reporting TD in some cases for reader’s reference.

### Effects of the inGAIT-VSO on ankle kinematics

The data and metrics extracted from the sensors of the inGAIT-VSO were useful to derive objective results related to the effects of the AFO stiffness on ankle kinematics, maximum DF and maximum PF achieved for the tested conditions of the 2mwt.

**Fig. 3:**
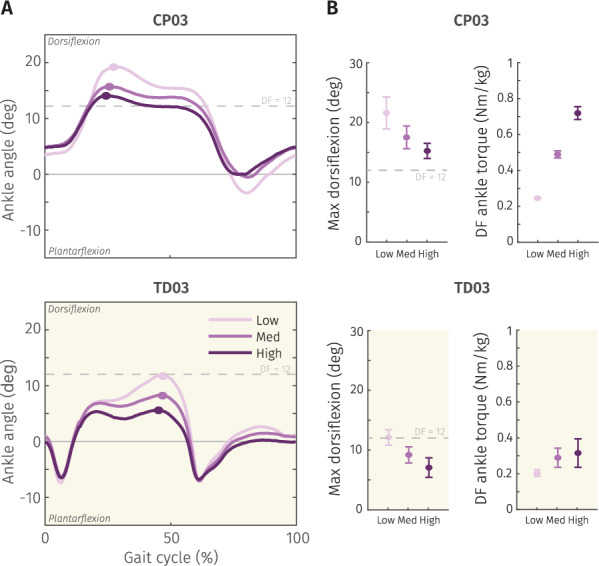
**A** Mean left ankle sagittal plane kinematics for the three tested conditions with the inGAIT-VSO during the 2mwt. Participant with CP (CP03, top) and a participant with TD (TD03, bottom). Light color (Low stiffness), mid color (Medium stiffness), dark color (High stiffness). **B** Mean and SD of maximum DF angles and maximum restoring torques associated to panel **A**

**Fig. 4:**
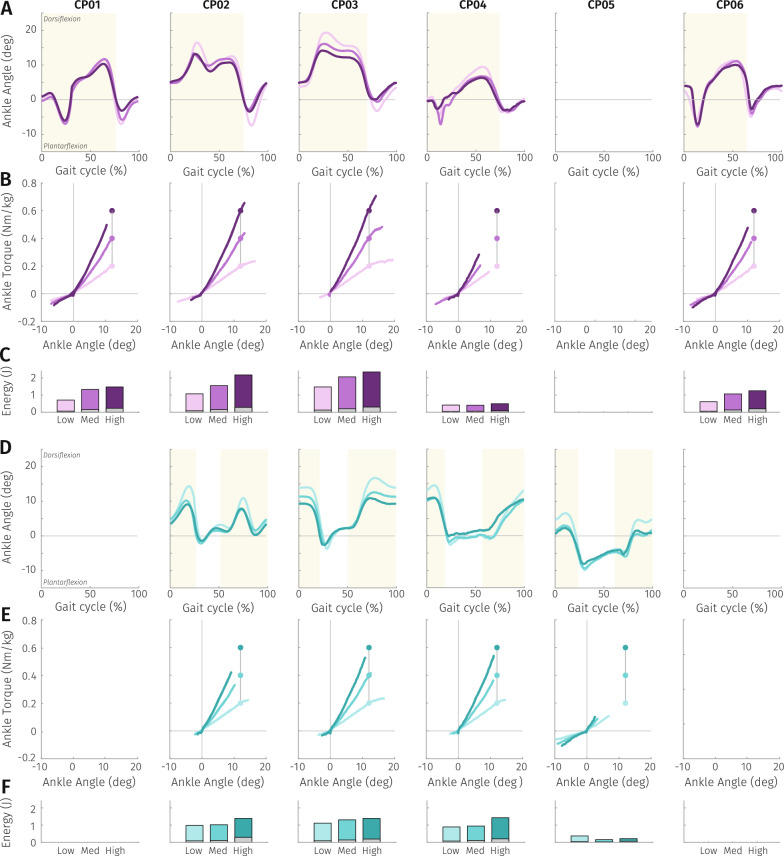
**A** and **D** Time-series of the mean ankle sagittal plane kinematics for the three tested conditions with the inGAIT-VSO in participants with CP. Purple (**A**) stands for left side, green (**D**) for right side. Gait cycles start with left heel strike, and stance phases are represented by the shaded yellow areas. Light, mid and dark line colors represent low, medium and high stiffness levels respectively. Participants with hemiparesia only wore the inGAIT-VSO on the affected side. **B** and **E**: Corresponding mean ankle angle-torque curves for the same tested conditions. The dots represent the desired torque at 12 deg of DF. **C** and **F**: mean energy towards DF stored (color) and mean energy towards DF loss (grey) for the conditions presented in panels **B** and **E**

Ankle sagittal plane kinematics were affected by the added stiffness of the inGAIT-VSO in both CP and TD participants (Fig. 3). As we expected, the device stiffness was inversely related to the maximum DF achieved by the participants with CP during the stance phase ($\chi ^2=16.22$, $p<0.01$, $DoF=26$), with an average decrease of 3 deg in maximum DF between Low and Med (post-hoc: $W=45$, $p<0.01$) , and 1 deg between Med and High (post-hoc: $W=44$, $p<0.01$). Maximum PF during swing was also significantly reduced with the increase of stiffness level ($\chi ^2=12.67$, $p<0.01$, $DoF=26$), but to a lesser degree than DF due to the design of the CAM, i.e. average difference of 1.4 deg between Low and Med (post-hoc: $W=4$, $p=0.03$) , and 1.5 deg between Med and High (post-hoc: $W=0$, $p<0.01$). Moreover, the generated graphs reveal the considerable level of “crouch gait” (larger ankle DF during the whole gait cycle) that participants with CP frequently exhibit compared to those with TD (Fig. 3). Specific pattern distinctions for each participant with CP can be observed in Fig. 4A and D. A complete overview of sensor-based metrics for all participants is presented in Appendix A.

### Stored energy

When selecting the three stiffness levels to be evaluated with the inGAIT-VSO, we defined them as the restoring torque to be achieved at a maximum DF of 12 deg (see Section "Experimental protocol"). This maximum DF was normally reached (with Low and Med stiffnesses) by most of the participants except CP05, (Fig. 4B, E). Although stiffer conditions led to a reduced DF, this reduction was outweighed by the increase in stiffness resulting in larger maximal restoring torque received (Fig. 4B, E).

The stored energy towards DF increased with the stiffness level, but also energy loss (Fig. 4C, F). Average energy efficiencies were 90.78$\%$, 88.06$\%$ and 83.39$\%$ for Low, Medium and High stiffnesses respectively, calculated for participants with CP. Individual energy data can be found in Appendix A.

### Effects of the inGAIT-VSO on participants’ performance

**Fig. 5:**
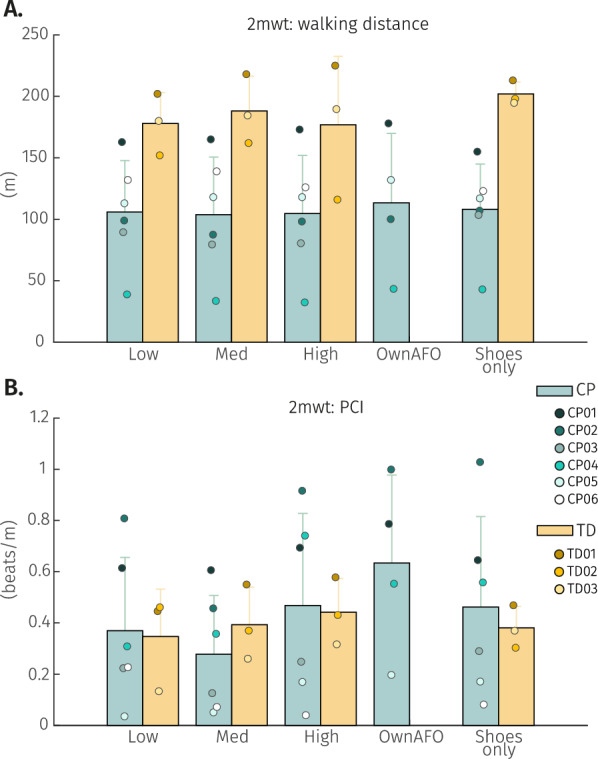
Group mean and SD for CP (green) and TD (yellow) participants performing the different walking conditions tested in the 2mwt. **A** represents the distance walked during the 2mwt, and **B** the PCI calculated. Individual participants’ responses are presented with color dots

The first-time use of the inGAIT-VSO did not reveal detrimental effects on the walking performance of the participants compared to their normal walking condition (i.e. shoes only or their own AFO if applicable). For participants with CP, averaged walked distances during the 2mwt were comparable among the different walking conditions (Fig. 5A). Comparison between their normal walking condition and the best performing stiffness with the inGAIT-VSO showed an average distance increase of 9.14$\%$, however, this difference was not significant ($W=4$, $p=0.22$). The testing order was randomized to prevent any order effect being misinterpreted as a stiffness effect.

Interestingly, although there was no significant difference between walking distances under different conditions for the 2mwt, there was a relevant PCI difference between these walking conditions (Fig. 5B). For participants with CP, this PCI difference was significant between the selected best performing stiffness with the inGAIT-VSO and their normal walking condition ($W=0$, $p<0.05$), with an average reduction of 45.26$\%$ when using the inGAIT-VSO.

The perceived effort values reported across the different walking conditions of the 2mwt typically fell within the range of “very, very light” to “fairly light” [26], with normally higher perceived effort reported by TD participants compared to CP participants under the same walking conditions (Appendix B). The outcome of the Borg’s scale did not result in a significant difference between the best performing stiffness selected for the inGAIT-VSO and the normal walking condition for participants with CP.

With respect to the tests performed on the second session of the study, participants with CP were marginally slower (6.11$\%$) and slightly more stable (2.62$\%$) with the inGAIT-VSO than in their normal walking condition during the 10mwt and the PBS test respectively. There was no significant difference for any of these metrics. More details can be found in Appendix B.

### Participants’ acceptance

**Fig. 6:**
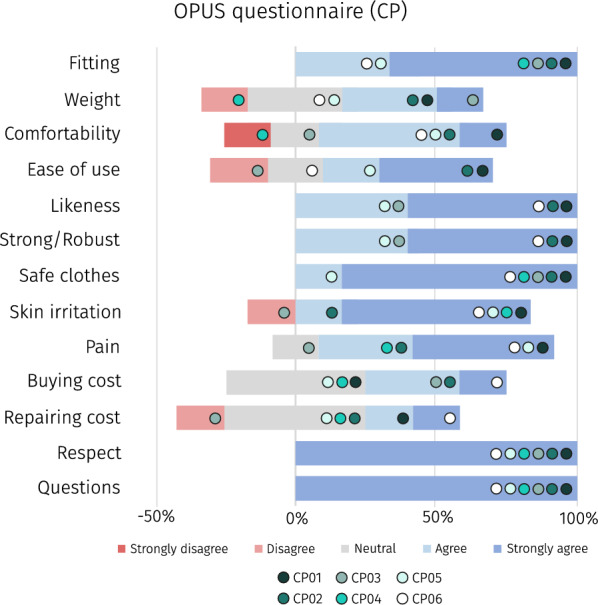
Participants with CP responses to the OPUS questionnaire. Respondents evaluated different features of the inGAIT-VSO on a Likert scale from 1—strongly disagree to 5—strongly agree

Overall, participants with CP were satisfied with their first use of the inGAIT-VSO, as their responses to the different features of the OPUS questionnaire were mostly positive (Fig. 6). Still, the weight of the AFO and the comfortability are reported features with potential to be improved in future re-designs.

Regarding the SUS scale completed in the second session, out of the CP participants, two participants scored the usability of the inGAIT-VSO as “excellent”, three as “good”, and one as “poor” (see Table 3).

**Table 3:** Rating interpretation for CP participants’ SUS scores

Participant ID	SUS score	Rating interpretation
CP01	82.50	Excellent
CP02	69.44	Good
CP03	57.14	Good
CP04	22.22	Poor
CP05	78.13	Excellent
CP06	72.50	Good

## Discussion

In this work, we used the inGAIT-VSO to investigate the effects of customizable AFO stiffness on the walking performance of children with CP. The objective was to assess whether the inGAIT-VSO has the potential to assess the influence of variable stiffness on pathological gait. In the study, six participants with CP (and three TD as pilot) performed different walking and balance tests with the inGAIT-VSO, and completed questionnaires related to acceptance and usability. The inGAIT-VSO allows customizable non-linear ankle stiffness and quick stiffness adjustments without the need for component replacement. Moreover, the device records data of participant’s walking performance that are useful to assess stiffness effects. Adjusting stiffness influenced participants kinematics differently depending on their gait, showing potential of variable-stiffness features and the importance of orthosis tailoring to individual needs [10, 11]. Overall, participants reported positive experiences with the device and good perception of its usability.

### Effects on participants’ kinematics

The inGAIT-VSO proved capable of influencing participants’ kinematics by modulating ankle stiffness across three discrete levels (Low, Medium and High). This modulation yielded objective data regarding participants’ walking performance, including ankle kinematics and energy storage. Specifically, increasing stiffness led to a significant reduction in both peak DF and PF kinematics across stiffness levels, acting during both swing and stance phases of gait. This is possible as the cam profile was engineered to operate bidirectionally with distinct non-linear stiffness for DF and PF respectively. The device most similar in functionality to the inGAIT-VSO is a speed-adaptive stiffness AFO recently developed by Hopkins et al. [17]. However, in their design, the action of the AFO is limited to the stance phase (DF) and their ankle torque–angle relationship is linear.

The effects of the inGAIT-VSO during the stance phase of walking were positive for children with crouch gait (i.e. CP02, CP03, CP04 in Fig. 4A, D), as evidenced by the reduced ankle DF from midstance onwards due to the resistance imposed by the spring-cam transmission. The decrease in DF was accompanied by an increased maximal restoring torque received. Thus, increasing the stiffness did not only correct the ankle kinematics (less DF—crouch—during stance), but the participants also received the intended assistance while using the inGAIT-VSO for all stiffness conditions (Fig. 4B, E). The kinematics of children with equinus (i.e. CP01 and CP06) only showed little changes between the different stiffness levels. Still, the restoring energy did increase with the level of stiffness and the patterns observed when using the inGAIT-VSO deviated from an equinus pattern towards a normal gait pattern (Fig. 4A, D). In the instance of CP05 (jump gait), it was observed that the ankle supported by the inGAIT-VSO (right) did not attain adequate DF during the stance phase. Experimenters noted a tendency in this participant to rely more on proximal joints, such as the hip, while walking, potentially due to the unfamiliarity with the inGAIT-VSO. This reliance on proximal joints may have contributed to the limited DF and consequently limited stored energy observed in this case for all levels of AFO stiffness.

During the swing phase, the inGAIT-VSO effectively mitigated drop-foot and foot slap occurrences in all participants, which is also considered an important function in AFO technology for CP [6]. These effects on participants’ kinematics with the inGAIT-VSO are in line with those of Kerkum et al. [7]. They demonstrated that varying the stiffness of a commercially available spring-hinged AFO had an effect during the stance phase not only on the ankle, but also on the knee kinematics, reducing crouch gait of children with CP. While our study did not directly evaluate knee joint kinematics as done by Kerkum et al., it is plausible that the effects observed with the inGAIT-VSO on the ankle are also accompanied with changes on the knee.

The potential of the inGAIT-VSO to influence gait kinematics in children with CP offers a promising avenue for evaluating the effects of various AFO stiffness configurations on walking performance. Nevertheless, our group observations only provide first indications on the effects of inGAIT-VSO in pathological CP gait patterns, and should be interpreted cautiously, as tests in a larger population are needed to draw definitive conclusions. Individualized orthotic alignment can already have a positive effect on pathological gait [34, 35], but other features such as tailored stiffness and user’s perspectives should also be considered to reach the optimal user’s walking performance [7, 10, 36]. Current literature highlights the challenges faced by healthcare professionals in prescribing orthotic management, lacking efficient tools to correlate AFO stiffness with individual needs [6, 10, 11]. By incorporating a customized torque–angle relationship within the inGAIT-VSO and directly assessing stiffness effects without requiring complex additional equipment, we anticipate that healthcare professionals can utilize our device as a valuable tool to guide their decisions in AFO prescription.

### Stored energy

The amount of net energy towards DF changed depending on the stiffness level in the three tested conditions (Fig. 4C, F). When the inGAIT-VSO was set to the Low stiffness condition, the stored energy was lower, reducing its ability to exert a substantial restoring torque. As the stiffness of the inGAIT-VSO increased, the stored energy increased and the device could exert a larger amount of restoring torque to the participant. It is important to note that this increase in stiffness comes at the expense of reduced range of motion. Furthermore, due to the low sample size of our study, we have not demonstrated that the increased energy release is directly related to a reduction in user’s energy cost. However, promising effects in that regard were found by others who evaluated a different passive mechanism for storing energy during stance on a larger sample size [16].

Computed energies may provide clinicians with valuable insights, as achieving a balance, individually tailored to each participant, between stiffness and range of motion is crucial to use the device to its potential. In contrast to our findings in energy differences for the three stiffness levels, Kerkum et al. [7] did not observe differences in energy return between a stiff (1.6 ± 0.4 Nm/deg) and a flexible condition (0.7 ± 0.2 Nm/deg) of their AFO. They attributed this lack of distinction to the insufficient disparity in stiffness between their conditions.

### Effects on participants’ performance

Compared to participants’ normal walking condition, the first use of the inGAIT-VSO did not adversely affect their walking performance (including distance, speed, perceived effort) or balance. Notably, within the CP population, a significantly lower PCI was observed when wearing the inGAIT-VSO compared to their normal walking condition (own AFO or shoes only). While similar studies have demonstrated that the use of AFOs reduces walking energy cost compared to shoes only, they have not identified differences across various AFO stiffness levels [7]. Nevertheless, our findings are preliminary and need further investigation for long-term exposure/use of the device. With respect to balance capabilities, we did not encounter significant differences between using the inGAIT-VSO and not using it. Conversely, Meyns et al. [36] proved that mediolateral gait stability is partly reduced by the stiffness of an AFO. It is noteworthy to mention that our assessment of balance was limited to static or short dynamic tasks from the PBS [30, 31], but we are not reporting balance capabilities during gait.

It is interesting that the participants in our study did not appear to perceive the reduced PCI associated with the use of the inGAIT-VSO. This observation is evidenced by their reported perceived effort using the Borg’s scale, which normally highlighted similar or lower values (less effort) for their normal walking condition (see Appendix B). This lack of benefit perception was previously identified by Medrano et al. [37], who found that humans cannot reliably perceive the metabolic benefits of today’s assistive exoskeletons. Surprisingly, the reported perceived effort was normally higher for TD participants compared to those with CP under the same walking conditions. This discrepancy could be attributed to the assumption that individuals with CP perceive their daily lives as more challenging, thus interpreting the effort required for performing any task differently. It is also likely that individuals with CP are more accustomed to walking with devices attached to the legs and their consequences.

### Usability

Mostly positive reports were received from the first experience with the inGAIT-VSO as seen in the OPUS questionnaire. However, although the mass of the inGAIT-VSO is comparable to similar solutions [15–17, 38], some participants with CP still identified weight as one of the key features to be improved. As for the SUS, a mean score of 65.66 ± 22.07 was obtained, slightly below the benchmark average of 68 [39], and lower than an actuated ankle exoskeleton designed for patients with CP (81.8) [38]. It is worth mentioning that five out of six participants with CP rated the inGAIT-VSO as either “good” or “excellent”, but CP04 reported a “poor” rating, which might be related to the use of a walker by this participant to perform the tests (limiting the use of the AFO). If we exclude this evaluation from the average, the SUS score would rise to 71.9.

### Study limitations

While studies that involve testing assistive robotic devices in CP commonly involve small cohorts [17, 38, 40] and although this study is exploratory in nature, we acknowledge that a first limitation of our investigation was the small sample size, so the results should be interpreted with caution. A second limitation of this study was that the determination of the best performing inGAIT-VSO condition relied on a newly introduced formula with no base on external sources. To the best of our knowledge, there is no study that investigates the relationship between (multi-factorial) walking performance variables and ankle stiffness to derive a best performing solution tailored to each participant. To address this gap, we decided to employ the proposed formula between sessions 1 and 2 using three variables rather than the 2mwt walking distance on its own. This decision was made because relying solely on the distance could be deemed incomplete as participants, after the first trial, had a reference of their previous distance and sometimes attempted to improve upon it regardless of the walking condition. A third concern is the possibility for the 2mwt to be too short to reach stabilized heart rates employed for the calculation of the PCI. However, it is important to note that the 2mwt has been established as a reliable and valid test for children with neuromuscular disorders [22]. Especially because of the repetition of different walking conditions along one session in our study, longer walking times could impose larger physical burden and challenges potentially unfeasible for this population. Furthermore, previous research has demonstrated that the PCI is a reliable metric in children with CP even when using non-steady-state heart rates [25], which suggest that the PCI over a 2-minute period can still provide meaningful results. A final limitation was that the device was tested in a controlled environment, which is common in exploratory studies [15–17, 38]. However, outcome measures, especially those related to the participants’ acceptance, may differ when used in daily living.

## Conclusion

Testing with the inGAIT-VSO showed that a variable-stiffness orthosis may be a promising option that specialists can use for quickly assessing a variety of conditions, assisting them in selecting an adequate/personalized stiffness level for a specific patient. The outcomes of this study serve as exploratory variables to guide a future larger randomized controlled trial, wherein the effectiveness of the inGAIT-VSO will be systematically evaluated in two ways: (1) as a clinical tool for healthcare professionals, and (2) as an assistive device for improving CP pathological gait in daily-life.

### Supplementary Information

Supplementary Material 1. Video showing the inGAIT-VSO working principle and an example of the tests with participants with CP.

## Data Availability

Important data is provided within the manuscript or supplementary information files. The rest of anonymized datasets generated and/or analyzed during the current study are available from the corresponding author on reasonable request.

## Appendix A

Table 4 presents the $mean \pm SD$ for max DF torque, maximum DF and maximum PF for all participants when using the inGAIT-VSO during the 2mwt. Table 5 presents the energy data computed for the same participants and trials.

**Table 4:** $Mean \pm SD$ of different metrics extracted from sensor data when participants used the inGAIT-VSO in three stiffness levels (Low, Med, High) during the 2mwt

Participant ID	Metric	inGAIT					
		Low		Med		High	
		Left	Right	Left	Right	Left	Right
TD01	Max DF torque (Nm/kg)	0.16 ± 0.04	0.22 ± 0.03	0.24 ± 0.08	0.43 ± 0.06	0.27 ± 0.10	0.68 ± 0.10
	Max DF (deg)	10.0 ± 3.1	14.0 ± 2.6	7.7 ± 2.2	13.7 ± 2.9	6.1 ± 2.0	13.9 ± 2.2
	Max PF (deg)	− 9.4 ± 4.0	-5.5 ± 3.4	− 7.4 ± 3.1	− 3.5 ± 2.6	− 9.0 ± 2.4	− 3.0 ± 2.1
TD02	Max DF torque	0.22 ± 0.02	0.15 ± 0.03	–	0.27 ± 0.07	0.68 ± 0.05	0.34 ± 0.09
	Max DF	14.5 ± 2.7	8.8 ± 1.9	–	8.6 ± 1.9	13.9 ± 1.3	7.5 ± 1.7
	Max PF	− 10.7 ± 3.9	− 11.0 ± 3.3	–	− 10.4 ± 2.6	− 7.3 ± 2.9	− 8.4 ± 2.4
TD03	Max DF torque	0.20 ± 0.02	0.20 ± 0.02	0.29 ± 0.05	0.33 ± 0.05	0.32 ± 0.08	0.41 ± 0.09
	Max DF	12.44 ± 1.5	12.0 ± 1.3	9.11 ± 1.22	10.2 ± 1.4	7.03 ± 1.5	8.9 ± 1.6
	Max PF	− 13.4 ± 2.0	− 10.7 ± 1.7	− 13.0 ± 2.1	− 10.9 ± 1.8	− 12.9 ± 1.7	− 9.7 ± 1.8
CP01	Max DF torque	0.21 ± 0.02	–	0.42 ± 0.04	–	0.54 ± 0.07	–
	Max DF	12.8 ± 1.7	–	12.7 ± 1.2	–	11.1 ± 1.1	–
	Max PF	− 11.3 ± 4.2	–	-9.1 ± 3.1	–	− 7.8 ± 3.6	–
CP02	Max DF torque	0.24 ± 0.01	0.23 ± 0.02	0.46 ± 0.03	0.39 ± 0.06	0.70 ± 0.04	0.54 ± 0.10
	Max DF	17.5 ± 2.4	16.5 ± 3.0	14.9 ± 1.6	12.1 ± 1.9	14.5 ± 1.3	11.2 ± 2.3
	Max PF	− 9.9 ± 3.7	− 3.8 ± 2.7	− 5.7 ± 3.6	− 4.8 ± 3.4	− 5.1 ± 2.5	− 3.8 ± 4.9
CP03	Max DF torque	0.25 ± 0.01	0.24 ± 0.01	0.49 ± 0.02	0.44 ± 0.03	0.72 ± 0.03	0.58 ± 0.06
	Max DF	21.38 ± 2.7	17.9 ± 2.2	17.51 ± 1.9	13.7 ± 1.6	15.24 ± 1.3	11.8 ± 1.3
	Max PF	− 5.5 ± 3.7	− 7.24 ± 3.0	− 2.3 ± 3.4	− 5.7 ± 2.5	− 0.8 ± 3.2	− 5.4 ± 3.1
CP04	Max DF torque	0.17 ± 0.02	0.22 ± 0.01	0.23 ± 0.02	0.38 ± 0.03	0.31 ± 0.03	0.57 ± 0.05
	Max DF	10.2 ± 1.1	15.12 ± 1.8	7.7 ± 0.5	11.39 ± 0.7	7.0 ± 0.7	11.55 ± 0.8
	Max PF	− 10.9 ± 4.1	− 3.4 ± 1.9	− 10.5 ± 2.9	− 2.5 ± 1.3	− 7.2 ± 2.5	− 0.49 ± 1.14
CP05	Max DF torque	–	0.14 ± 0.03	–	0.12 ± 0.03	–	0.17 ± 0.06
	Max DF	–	8.7 ± 1.8	–	4.3 ± 0.9	–	3.9 ± 1.7
	Max PF	–	− 13.1 ± 2.2	–	− 12.4 ± 1.6	–	− 10.5 ± 1.4
CP06	Max DF torque	0.20 ± 0.01	–	0.38 ± 0.03	–	0.50 ± 0.04	–
	Max DF	11.9 ± 0.9	–	11.5 ± 0.8	–	10.4 ± 0.6	–
	Max PF	− 10.5 ± 1.2	–	− 9.4 ± 1.7	–	− 7.8 ± 1.4	–

**Table 5:** Mean energy stored (loading), released (unloading) and loss computed for the left and right inGAIT-VSO when being used in three different stiffness levels (Low, Med, High) during the 2mwt

Participant ID	Side	Energy stored (J)			Energy released (J)			Energy loss (J)		
		Low	Med	High	Low	Med	High	Low	Med	High
TD01	Left	0.58	0.64	0.60	0.52	0.54	0.46	0.06	0.10	0.14
	Right	1.07	2.02	3.14	0.98	1.76	2.57	0.09	0.26	0.57
TD02	Left	0.75	–	1.92	0.68	–	1.67	0.07	–	0.25
	Right	0.30	0.50	0.51	0.26	0.44	0.41	0.03	0.06	0.10
TD03	Left	0.69	0.68	0.60	0.63	0.59	0.49	0.06	0.09	0.11
	Right	0.64	0.86	0.97	0.58	0.75	0.80	0.06	0.11	0.17
CP01	Left	0.71	1.33	1.47	0.65	1.17	1.24	0.06	0.16	0.23
	Right	–	–	–	–	–	–	–	–	–
CP02	Left	1.07	1.55	2.17	0.99	1.39	1.89	0.09	0.16	0.28
	Right	0.98	1.02	1.38	0.89	0.91	1.09	0.08	0.11	0.29
CP03	Left	1.47	2.06	2.35	1.35	1.86	2.05	0.13	0.20	0.30
	Right	1.11	1.31	1.38	1.02	1.17	1.20	0.09	0.14	0.19
CP04	Left	0.42	0.41	0.50	0.37	0.36	0.42	0.04	0.05	0.08
	Right	0.89	0.95	1.43	0.82	0.84	1.23	0.08	0.11	0.20
CP05	Left	–	–	–	–	–	–	–	–	–
	Right	0.37	0.16	0.21	0.32	0.13	0.16	0.04	0.03	0.06
CP06	Left	0.61	1.07	1.25	0.56	0.93	1.05	0.06	0.13	0.21
	Right	–	–	–	–	–	–	–	–	–

## Appendix B

Tables 6 and 7 present the main metrics registered during the tests performed in sessions 1 and 2 respectively.

**Table 6:** No-sensor metrics registered during the 2mwt

Participant ID	Metric	inGAIT			Own AFO	Shoes only
		Low	Med	High		
TD01	Distance (m)	202.0	218.0	225.0	–	213.0
	PCI (beats/m)	0.45	0.55	0.58	–	0.47
	Borg	13	12	14	–	11
TD02	Distance	152.0	162.0	116.0	–	198.0
	PCI	0.46	0.37	0.43	–	0.30
	Borg	12	12	13	–	11
TD03	Distance	180.0	184.5	189.6	–	194.7
	PCI	0.13	0.26	0.32	–	0.37
	Borg	10	10	8	–	8
CP01	Distance	162.8	165.0	173.0	178.0	155.0
	PCI	0.61	0.61	0.69	0.79	0.65
	Borg	12	10	9	6	10
CP02	Distance	99.0	87.5	98.2	100.1	107.0
	PCI	0.81	0.46	0.92	1.00	1.03
	Borg	7	8	8	7	8
CP03	Distance	89.5	79.5	80.5	–	103.5
	PCI	0.22	0.13	0.25	–	0.29
	Borg	6	8	8	–	6
CP04	Distance	38.9	33.6	32.4	43.4	43.0
	PCI	0.31	0.36	0.74	0.55	0.56
	Borg	6	10	12	6	6
CP05	Distance	113.0	118.0	118.0	132.0	117.0
	PCI	0.04	0.05	0.17	0.20	0.17
	Borg	10	8	11	9	8
CP06	Distance	132.0	139.0	126.0	–	123.0
	PCI	0.23	0.07	0.04	–	0.08
	Borg	10	9	11	–	12

**Table 7:** No-sensor metrics registered during the 10mwt and the PBS

Participant ID	Metric	inGAIT	OwnAFO	Shoes only
TD01	Speed (m/s)	2.04	–	2.01
TD02	Speed	1.93	–	1.52
TD03	Speed	1.82	–	1.74
CP01	Speed	1.68	1.65	–
	PBS total	51.0	49.5	–
	PBS static	23.0	21.5	–
CP02	Speed	1.18	1.16	–
	PBS total	36.5	40.0	–
	PBS static	17.0	18.5	–
CP03	Speed	0.71	–	0.71
	PBS total	38.0	–	38.5
	PBS static	15.0	–	15.5
CP04	Speed	0.45	0.68	–
	PBS total	22.0	18.5	–
	PBS static	8.0	7.0	–
CP05	Speed	1.04	1.38	–
	PBS total	50.5	49.5	–
	PBS static	24.5	22.5	–
CP06	Speed	1.43	–	1.21
	PBS total	56.0	–	55.0
	PBS static	28.0	–	27.0

## Abbreviations

AFO: Ankle-foot orthosis
CP: Cerebral palsy
TD: typical development
VSO: Variable stiffness orthosis
DF: dorsiflexion
PF: plantarflexion
FSR: force sensitive resistors
HNJ: Hospital Niño Jesús
2mwt: two minutes walking test
10mwt: ten meters walking test
PCI: Physiological cost index
PBS: Pediatric balance scale
